# 3D Organon VR Anatomy

**DOI:** 10.5195/jmla.2022.1341

**Published:** 2022-04-01

**Authors:** Jason Lilly

**Affiliations:** 1 jlilly1@iuhealth.org, Systems Librarian, IU Health Medical Library, Indiana University Health, Indianapolis, IN

## Abstract

**3D Organon VR Anatomy.** Medis Media Pty Ltd, 2/23 Illawong Str., Surfers Paradise, Gold Coast, Queensland 4217, Australia; https://www.3dorganon.com/; prosupport@3dorganon.com; +61.4.5190.5904; individual and institutional subscriptions, contact for pricing.

## INTRODUCTION AND BACKGROUND

Before launching into the review of 3D Organon VR Anatomy, I feel it would be helpful to provide some background information on virtual reality (VR), the hardware involved, and my personal experience using VR in the arena of medical libraries and medical education. What exactly is virtual reality? I particularly like this definition from the Virtual Reality Society, which describes VR as

a three-dimensional generated environment which can be explored and interacted by a person. That person becomes a part of that virtual world or is immersed within this environment and whilst there, is able to manipulate objects or perform a series of actions [1].

The key factor in this definition is the idea of immersion, which separates virtual reality from other forms of “reality” such as augmented or mixed. These add virtual elements to your physical reality but do not immerse you fully in a virtual realm. The virtual reality headset, which in technology parlance is often referred to as a head-mounted display (HMD), completely dominates your field of vision and, with attached headphones, your sense of hearing, thus providing a truly immersive experience. It is that immersive aspect that has made VR a powerful tool in medicine, for example treating combat veterans with PTSD [2] or helping burn patients with pain management during treatment [3].

Oculus by Facebook [4] and Vive by HTC [5] continue to be the major players in the VR landscape since they first introduced headsets to the market circa 2016; however, there is now a whole line of headsets from other companies that are compatible with Windows 10. Oculus has moved away from PC-powered VR in recent years to focus on their stand-alone VR headset, the Quest. PC-powered VR is still the higher-end experience based on utilizing a high-level graphics card and the processing power of a gaming computer; however, in terms of price range for a virtual reality station, it could fall between $2,500 and $5,000, depending on the machine purchased and peripherals. Pricing for the Quest 2 starts at $299, and it is a quality VR experience. I have personal experience using the HTC Vive and Vive Pro, the Oculus Rift, and the Oculus Quest and Quest 2.

My experience with virtual reality dates to the early 1990s when the company Virtuality [6] set up a demo of their game *Dactyl Nightmare* at the student union while I was a student at Miami University in Ohio. At that point in time, the processing power was not sufficient to make VR a commercially viable product. VR in 64-bit graphics just does not compare to the VR technology of today. In March 2017, I was able to attend South by Southwest, and a major theme of the conference was virtual reality's use and impact in medicine, which reignited my passion for VR in connection with my role in medical libraries. In October 2017, the Ruth Lilly Medical Library technology team was invited by NNLM to speak at the Midwest Chapter and MHSLA conference [7]. My part of the presentation focused on VR in medical practice. An article published in 2018 details the RLML tech team's early days with VR and 3D printing [8]. The *JMLA* virtual project published in 2019 details my role in the creation of the Nexus Virtual Reality Lab at the Ruth Lilly Medical Library [9]. Since mid-2017, I have introduced hundreds of medical students, faculty, and staff to VR for the first time. In terms of exploring the landscape continuously for useful VR programs, 3D Organon VR Anatomy has always been the go-to showcase program for me to demonstrate how this technology could be utilized in medical education.

## 3D ORGANON VR ANATOMY

3D Organon VR Anatomy was featured by Mark Zuckerberg in his keynote address at the Oculus Connect 3 conference in 2016 and has won numerous awards [10]. The company Medis Media, which produces 3D Organon, was formed by Dr. Athanasios Raikas and Dr. Panaigoti Kordali, anatomy instructors at Bond University in Australia [11]. Their knowledge of anatomy, along with a team of highly skilled programmers, forms the backbone of the program as reflected by its quality. According to a brochure provided by the company, 3D Organon is broken into fifteen human body systems with more than 10,000 realistic anatomical structures, over 550 action modules of muscles and organs, cadaveric images, and microscopic anatomy models. The true power of the program lies in being able to virtually inspect each anatomical structure, turning it and manipulating it to see it from all angles, and being able to see how the structures connect within each system in this truly immersive environment. In terms of my experience with medical students, the 3D aspect really helps students grasp what the structure is, where it fits, and its role within the system. For each structure, there is a detailed description. There is the ability to fade out structures so a student can really focus on its individual placement within the system. The program is also available in fifteen languages and includes recording options for instructors to share video with students from the program as well as drawing modes to help highlight structures. The interface and action with the VR controllers is highly intuitive, and it does not take long for a user to get comfortable with the program. The installation process for PC-powered VR is simple; however, the Oculus Quest installation process is more difficult because it is not available in the Oculus store. The company does provide detailed installation instructions, and they offer a great deal of content on their YouTube channel that certainly helps to visualize what the program can do [12].

The program offers different pricing tiers based on the platform and added features. There are prices listed on their website for individual use licenses, but for institution pricing the company must be contacted for a tailored quote [13]. They follow a twelve-month subscription model. The least expensive option is for stand-alone VR like the Oculus Quest 2. There are a standard and a premium version for PC-powered VR. The premium version includes additional content like USMLE testing modules. Depending on the VR headset you are using with the PC-powered option, there are additional augmented reality features where you can overlap the VR image over an actual person. The company has expanded its offerings into different formats, but the true strength of the program is that it is one of a kind in the VR realm.

## COMPARISONS

It is difficult to conceive of direct comparisons for 3D Organon in the VR space. Complete Anatomy by 3D4Medical is a popular anatomy program utilized by students, but it is not available for VR. Being able to manipulate structures and view them from any angle is certainly a strong selling point in the immersive environment of 3D Organon [14]. It does appear that 3D4Medical is dipping their toes into mixed reality with their program Holohuman, and it works with the Microsoft Hololens, but I have no direct experience with it [15]. Primal Picture's Anatomy TV has a VR component now, but I have never found their 3D functionality very user friendly [16]. The controls and functionality of 3D Organon are natural and user friendly in comparison, with the program having been designed specifically for VR. You by ShareCare is an impressive VR program in terms of its visualizations and is certainly a program I recommend based on it being inexpensive; however, it lacks the breadth of information available in 3D Organon and its user interface is quite complex [17]. In terms of VR programs that are applicable to medical education, 3D Organon is the best I have encountered.

## USE CASES

I have been in contact with the company, and they provided a listing of US libraries that have used 3D Organon. These include Western Carolina University Hunter Library, Eastern Carolina University William E. Laupus Health Sciences Library, California State University Fullerton, and Temple University Ginsburg Health Sciences Library. I am aware of local institutional use in the Indianapolis area by the Ruth Lilly Medical Library and Marian University. The company lists multiple international use cases on their website. There is still a relatively inexpensive version of the program available on the Steam platform and the Oculus store. The company was clear that this version is intended for personal use and having it installed on machines accessible to multiple users would be a violation of the license agreement. This was the version that I was using to demonstrate the program. It serves as an example that as these companies grow, they are learning to monetize their products more effectively. In my experience, most VR programs are licensed for single use without consideration for multi-user situations like a library. The work-around had been to buy one license for each machine the program was installed on. This is an evolving issue with VR applications.

The best use case I am personally aware of for the implementation of 3D Organon in medical education was conducted by Debra Patterson, assistant professor of clinical and imaging sciences at the Indiana University School of Medicine [18]. During the fall semester of 2019 and into the spring semester of 2020, Patterson incorporated 3D Organon labs into her undergraduate classes for medical imaging. The first lab was structured to familiarize the students with the VR equipment and the program itself. The second had them use the program to answer specific questions about the external jugular vein. Based on specific student feedback such as “I would like if each week we had a different anatomy section we could explore and make our own anatomy assignment in a way,” Patterson allowed more freedom in the third assignment for the students to choose anatomy in the program to describe, and the student response was highly positive: “I enjoy being able to choose anatomy I am interested in and describing how it is important to MRI.” However, like so many things in 2020, her plans for the remainder of that academic year were disrupted by COVID-19 as classes were converted to virtual. Patterson has expressed excitement about building on her previous attempt and incorporating 3D Organon into future classes based on the positive student reaction.

## CONCLUSION

We are currently seeking grant funding to establish a technology lab at the IU Health Medical Library, which would include VR stations and Oculus Quest 2s for department checkout. 3D Organon VR Anatomy is certainly a program that would be incorporated in this plan if funding is secured. The program is a true showcase of what VR can do in the medical field and a great software to demo when trying to get students, staff, and faculty excited about virtual reality.

## References

[1] What is virtual reality? [Internet]. Virtual Reality Society; 2017 [cited 15 Jun 2021]. <https://www.vrs.org.uk/virtual-reality/what-is-virtual-reality.html>.

[2] Rizzo A, Shilling R. Clinical virtual reality tools to advance the prevention, assessment, and treatment of PTSD. Eur J Psychotraumatol. 2017 Jan 16;8(sup5):1414560. DOI: 10.1080/20008198.2017.1414560. PMID: 29372007; PMCID: 29372007. 29372007 PMC5774399

[3] Scapin S, Echevarría-Guanilo ME, Boeira Fuculo Junior PR, Gonçalves N, Rocha PK, Coimbra R. Virtual reality in the treatment of burn patients: a systematic review. Burns. 2018 Sep;44(6):1403–16. DOI: 10.1016/j.burns.2017.11.002. PMID: 29395400. 29395400

[4] Oculus from Facebook [Internet]. Facebook Technologies, LLC [cited 15 Jun 2021]. <https://www.oculus.com/>.

[5] VIVE United States: Discover virtual reality beyond imagination [Internet]. VIVE [cited 15 Jun 2021]. <https://www.vive.com/us/>.

[6] Virtuality (product) [Internet]. Wikipedia. Wikimedia Foundation; 2021 [cited 15 Jun 2021]. <https://en.wikipedia.org/wiki/Virtuality_(product)>.

[7] Midwest MLA Tech Topic – Realizing medicine: how virtual reality and augmented reality are impacting medical practice and education [Internet]. Midwest Matters | The blog of NNLM Region 6. 2017 [cited 15 Jun 2021]. <https://news.nnlm.gov/region_6/2017/11/midwest-mla-tech-topic-realizing-medicine-how-virtual-reality-and-augmented-reality-are-impacting-medical-practice-and-education/>.

[8] Lilly J, Kaneshiro K. Librarians dream of electric cats: a tech team's journey into the world of emerging technologies. Against the Grain. 2018;30(4). DOI: 10.7771/2380-176X.8278.

[9] Lilly J, Kaneshiro KN, Misquith C, Dennett B. Creating a new “reality” for medical education: the Nexus Reality Lab for virtual reality. J Med Libr Assoc. 2019 Oct;107(4):609–10. DOI: 10.5195/jmla.2019.784. PMID: 31607823; PMCID: 31607823. 31607823 PMC6774549

[10] Mark Zuckerberg on the future of education at OC3 features 3D Organon VR Anatomy [Internet]. YouTube; 2016 [cited 15 Jun 2021]. <https://youtu.be/HxDIRgEAZ1I>.

[11] Mosca D. PRESS RELEASE: 3D Organon, the most advanced extended reality (XR) medical anatomy platform. Great tool for remote delivery teaching. [Internet]. Media Database; 2020 [cited 15 Jun 2021]. <https://getthewordout.com.au/press-release/press-release-3d-organon-the-most-advanced-extended-reality-xr-medical-anatomy-platform-great-tool-for-remote-delivery-teaching/>.

[12] 3D Organon [Internet]. YouTube [cited 15 Jun 2021]. <https://www.youtube.com/channel/UC6BE95PRVcikYCKCqa3tiBw>.

[13] 3D Organon Anatomy: Professional [Internet]. 3D Organon [cited 15 Jun 2021]. <https://store.3dorganon.com/>.

[14] Complete Anatomy - advanced 3D anatomy platform [Internet]. Complete Anatomy; 2021 [cited 15 Jun 2021]. <https://3d4medical.com/>.

[15] HoloHuman - 3D4Medical [Internet]. Complete Anatomy; 2021 [cited 15 Jun 2021]. <https://3d4medical.com/apps/holohuman>.

[16] Primal VR [Internet]. Primal Pictures; 2021 [cited 15 Jun 2021]. <https://www.primalpictures.com/2020/08/05/primal-vr/>.

[17] Sharecare YOU. [cited 15 Jun 2021]. <https://www.sharecareyou.com/>.

[18] Misquith C, Patterson D. Using VR to enhance anatomy education for medical imaging learners [Internet]. IUPUI; 2020 [cited 15 Jun 2021]. <https://scholarworks.iupui.edu/handle/1805/22748>.

